# Pulsed field ablation for paroxysmal atrial fibrillation and atrial flutter in a patient with dextrocardia and prior secundum atrial septal defect repair: a case report

**DOI:** 10.1093/ehjcr/ytag599

**Published:** 2026-08-11

**Authors:** Assel Chinybayeva, Moldir Yerikkyzy, Zhanasyl Suleymen, Elmira Yessimbekova, Ayan Abdrakhmanov

**Affiliations:** Heart Rhythm Research Institute, NJSC ‘Astana Medical University’, 49A Beibitshilik street, Astana 010000, Kazakhstan; Heart Rhythm Research Institute, NJSC ‘Astana Medical University’, 49A Beibitshilik street, Astana 010000, Kazakhstan; Heart Rhythm Research Institute, NJSC ‘Astana Medical University’, 49A Beibitshilik street, Astana 010000, Kazakhstan; Heart Rhythm Center, National Coordination Center for Emergency Medicine, 14 Hussein bin Talal street, Astana 010000, Kazakhstan; Heart Rhythm Research Institute, NJSC ‘Astana Medical University’, 49A Beibitshilik street, Astana 010000, Kazakhstan; Heart Rhythm Research Institute, NJSC ‘Astana Medical University’, 49A Beibitshilik street, Astana 010000, Kazakhstan; Heart Rhythm Center, National Coordination Center for Emergency Medicine, 14 Hussein bin Talal street, Astana 010000, Kazakhstan

**Keywords:** Pulsed field ablation, Electroporation, Dextrocardia, Atrial fibrillation, Atrial flutter, Case report

## Abstract

**Background:**

Pulsed field ablation (PFA) is a novel non-thermal ablation modality that has demonstrated efficacy and safety in the treatment of common arrhythmias such as atrial fibrillation (AF) and atrial flutter (AFL). However, evidence regarding its use in patients with dextrocardia remains scarce.

**Case summary:**

A 63-year-old woman with dextrocardia and situs in vs. totalis, and with prior surgical atrial septal defect (ASD) repair, presented with heart palpitations that did not respond to antiarrhythmic drugs. Ambulatory and Holter monitoring documented short episodes of AF and AFL. Computed tomography (CT) of the heart and its structures before the procedure enabled anatomical orientation and planning of the procedure. Catheter ablation was performed using a Pentaspline PFA system (FARAPULSE, Boston Scientific) under fluoroscopic guidance without electroanatomical mapping. Pulmonary vein isolation, along with ablation of the left atrial roof, posterior wall of the left atrium, and cavotricuspid isthmus, was successfully achieved. The procedure was successful, and no recurrence of arrhythmia was observed on 6-month Holter monitoring.

**Discussion:**

This case demonstrates that combined PFA for AF and AFL is feasible in patients with complex congenital anatomy, including dextrocardia following ASD repair. Mirror-image cardiac anatomy presents unique challenges for catheter navigation and anatomical orientation. Pre-procedural cardiac CT was essential for procedural planning and accurate spatial orientation, facilitating successful PFA in this anatomically complex setting.

Learning points

- Pulsed field ablation may be a feasible and safe strategy for treating atrial fibrillation and atrial flutter in patients with dextrocardia, situs in vs. totalis and prior surgical atrial septal defect repair.
- Pre-procedural cardiac computed tomography is essential for understanding mirror-image anatomy and facilitating procedural planning in complex congenital settings.

## Introduction

Pulsed field ablation (PFA) is an emerging, predominantly non-thermal catheter-based ablation technique that induces myocardial injury by irreversible electroporation.^1,2^ There are limited data on the use of PFA in patients with dextrocardia, with previously reported cases primarily focusing on atrial fibrillation (AF). To our knowledge, the application of PFA for atrial flutter (AFL) in this population has not yet been determined. We describe a case of combined AF and AFL ablation in a patient with dextrocardia and prior atrial septal defect (ASD) repair.

## Summary figure

**Figure:**
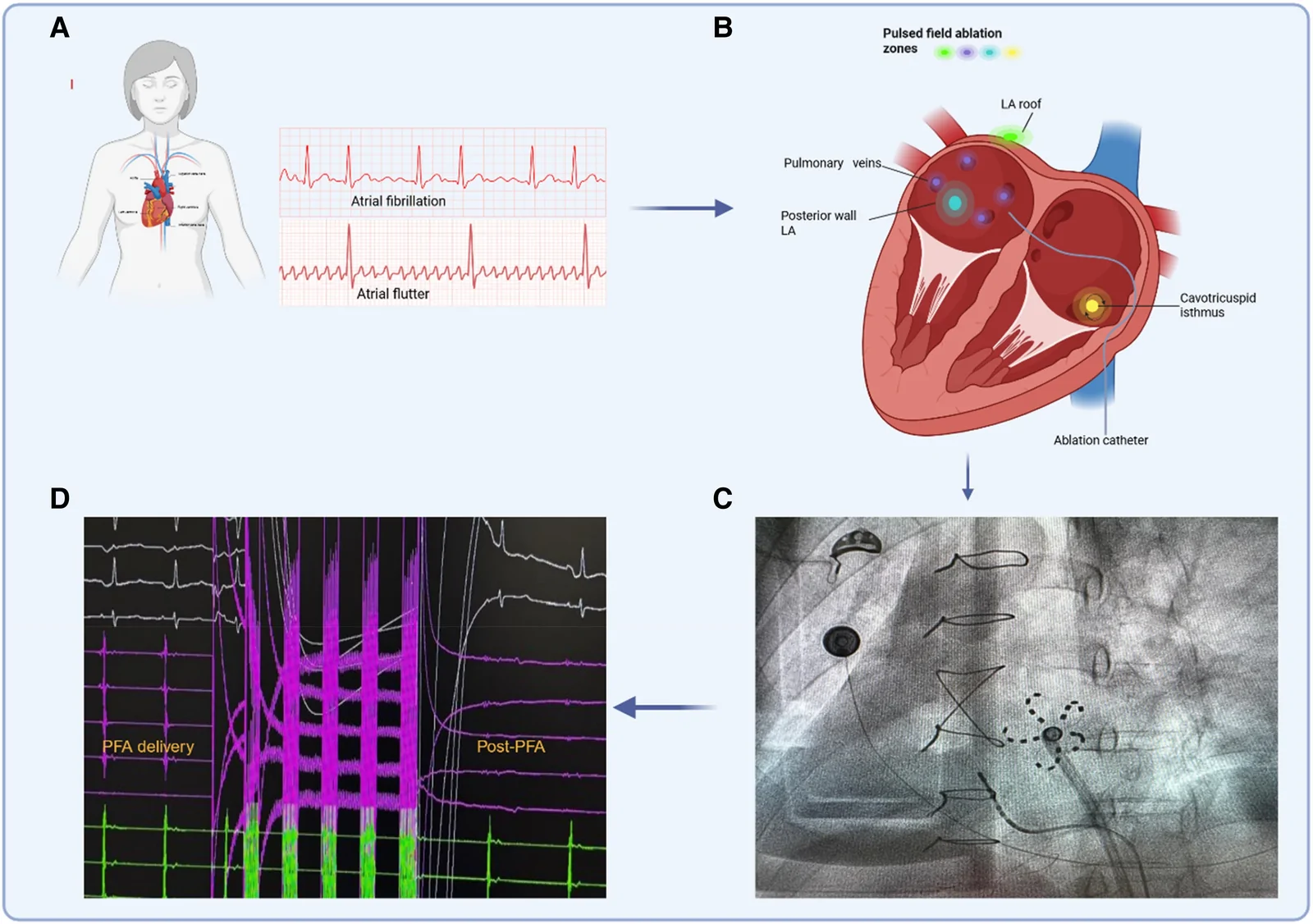
Pulsed field ablation for paroxysmal atrial fibrillation (AF) and atrial flutter (AFL) in dextrocardia. (*A*) A 63-year-old woman with palpitations and situs in vs. totalis presented with paroxysmal AF and AFL on electrocardiography. (*B*) PFA was performed, including pulmonary vein isolation, posterior wall and left atrial roof, and cavotricuspid isthmus ablation. (*C*) Farawave catheter (Boston Scientific) was used. (*D*) A total of 50 applications were delivered, achieving acute procedural success without complications.

## Case presentation

A 63-year-old female patient with known dextrocardia and situs in vs. totalis was admitted to the hospital due to palpitations. The medical history of the patient included surgical repair of a secundum ASD with an autologous pericardial patch under cardiopulmonary bypass (2014), alongside mild arterial hypertension. Following the surgical procedure, she developed paroxysmal AF. However, over the past 2 months, her symptoms had worsened and become refractory to pharmacological therapy.

**Figure 1:**
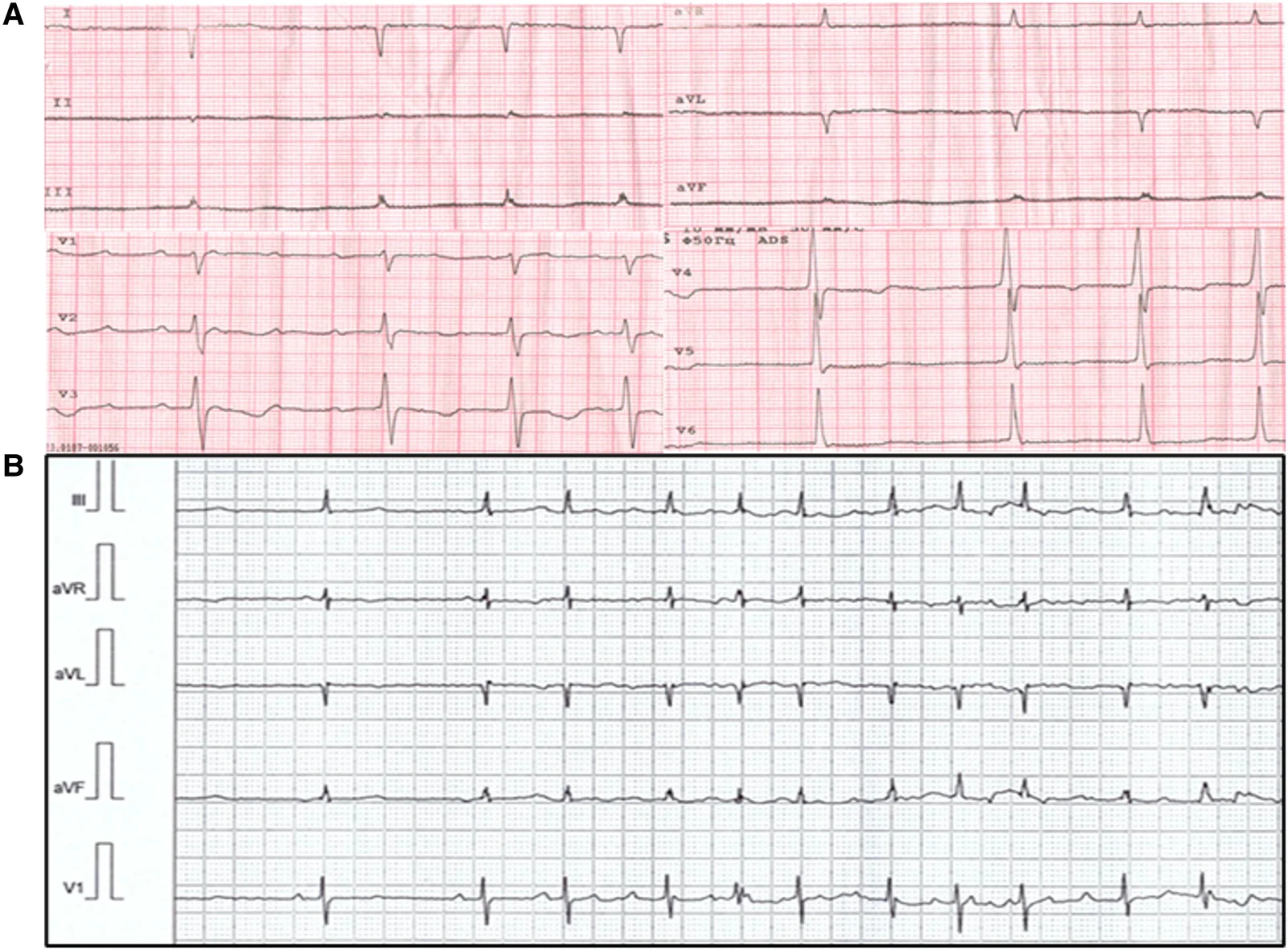
Electrocardiographic findings: standard electrocardiogram and Holter monitoring. (*A*) Ambulatory electrocardiogram. Atrial flutter rhythm with a heart rate of 85–100 beats per minute and 2:1 atrioventricular conduction. Due to dextrocardia, a positive QRS complex is observed in aVR. Paper speed: 50 mm/s; calibration: 10 mm/mV. (*B*) Ambulatory Holter electrocardiogram. Episode of paroxysmal atrial fibrillation. Paper speed: 25 mm/s; calibration: 10 mm/mV.

Before admission, the patient underwent outpatient coronary computed tomography (CT) angiography after the detection of inverted T-waves on the electrocardiogram (ECG). The imaging excluded obstructive coronary artery disease. Also, an outpatient ECG revealed paroxysmal AFL (*Figure 1A*), whereas a Holter ECG reported a short episode of AF (*Figure 1B*). A timeline summarizing the key clinical events is presented in Supplementary material online, *Table S1*.

**Figure 2:**
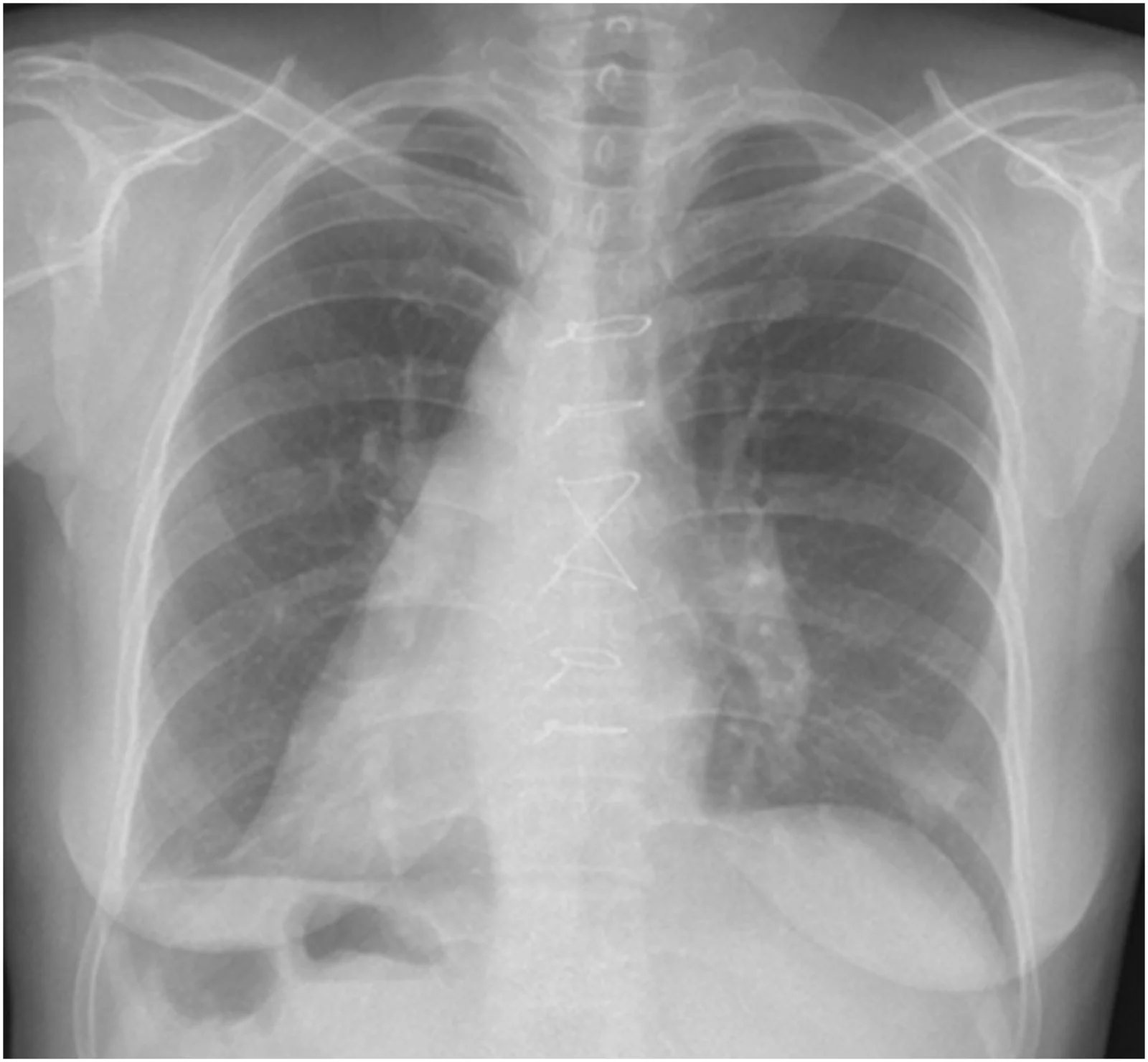
Chest X-ray. Post-sternotomy changes are noted. Dextrocardia of the heart is present.

Given the presence of dextrocardia and situs in vs. totalis, a comprehensive diagnostic evaluation was performed: chest X-ray (*Figure 2*) and transoesophageal echocardiography (TEE). The latter confirmed the following: an intact atrial septal patch with no residual shunting; no evidence of thrombi in the left atrial appendage or cardiac chambers; mild mitral regurgitation and minimal tricuspid insufficiency were noted; ejection fraction was 60%.

**Figure 3:**
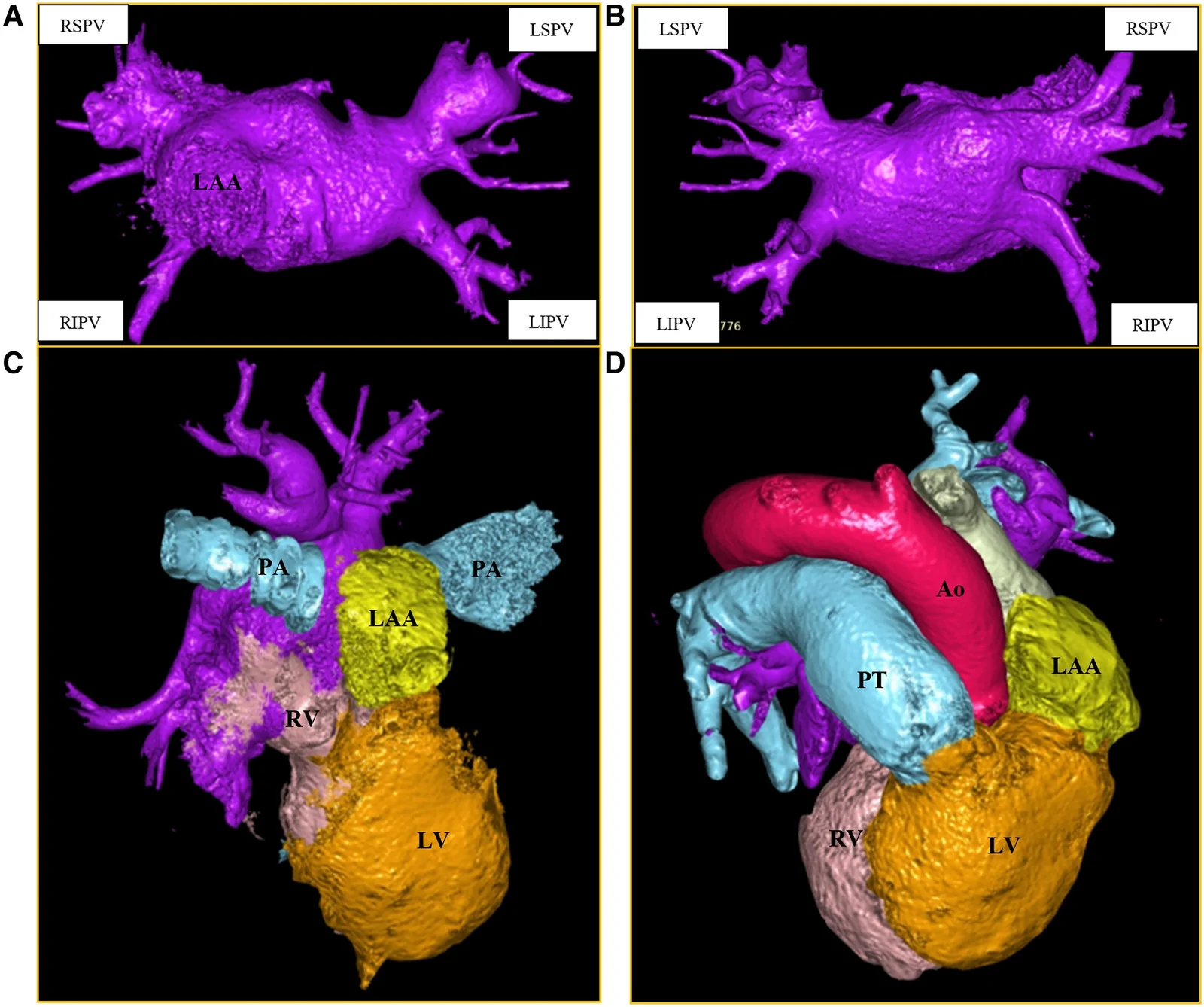
Computed tomography with 3D reconstruction. (*A*, *B*) Pulmonary veins in anterior (*A*) and posterior (*B*) views. (*C*, *D*) Heart in lateral (*C*) and anterior (*D*) views, demonstrating dextrocardia. SPV, superior pulmonary vein; IPV, inferior pulmonary vein; PA, pulmonary artery; LAA, left atrial appendage; RV, right ventricle; LV, left ventricle; PT, pulmonary trunk; Ao, Aorta.

The procedure was performed under general anaesthesia using fluoroscopy and FARAPULSE (Boston Scientific) system. Pre-procedural contrast-enhanced cardiac CT with multiplanar reconstructions to better navigate the mirror-image anatomy was implemented (*Figure 3*).

Due to the patient’s small body habitus (BSA-1.63 m^2^) and narrow vessel diameter, a 5F 10-pole catheter was used for coronary sinus (CS) cannulation through the femoral vein, as the 6F catheter could not be advanced. A 62 cm long Swartz introducer was placed in the right atrium.

To reduce the risk of coronary vasospasm, 3 mg of nitroglycerin was injected into the right atrial cavity before ablation. As a result, no signs of coronary artery spasm were observed during ablation in the isthmus, where such spasm is a known risk associated with procedures in this region. A transseptal puncture (TSP) was performed under fluoroscopic guidance without additional imaging [TEE/ICE (intracardiac imaging)] using a mirror-image approach adapted to the patient’s reversed cardiac anatomy, with inversion of standard fluoroscopic projections (left anterior oblique instead of right anterior oblique and vice versa). Pre-procedural cardiac CT imaging also facilitated anatomical orientation in the setting of prior ASD patch repair. Accordingly, transseptal access was successful without any technical difficulties.

The patient received apixaban 10 mg/day for 1 month before and 3 months after the ablation. During the procedure, 8000 IU of intravenous heparin were administered, so the activated clotting time was maintained between 300 and 400 s. Following standard measures to prevent air embolism, the Preface TSP introducer was exchanged for a Faradrive 13Fr steerable introducer. A Farawave 12Fr, 31 mm ablation catheter was advanced into the left atrium through the Faradrive introducer.

**Figure 4:**
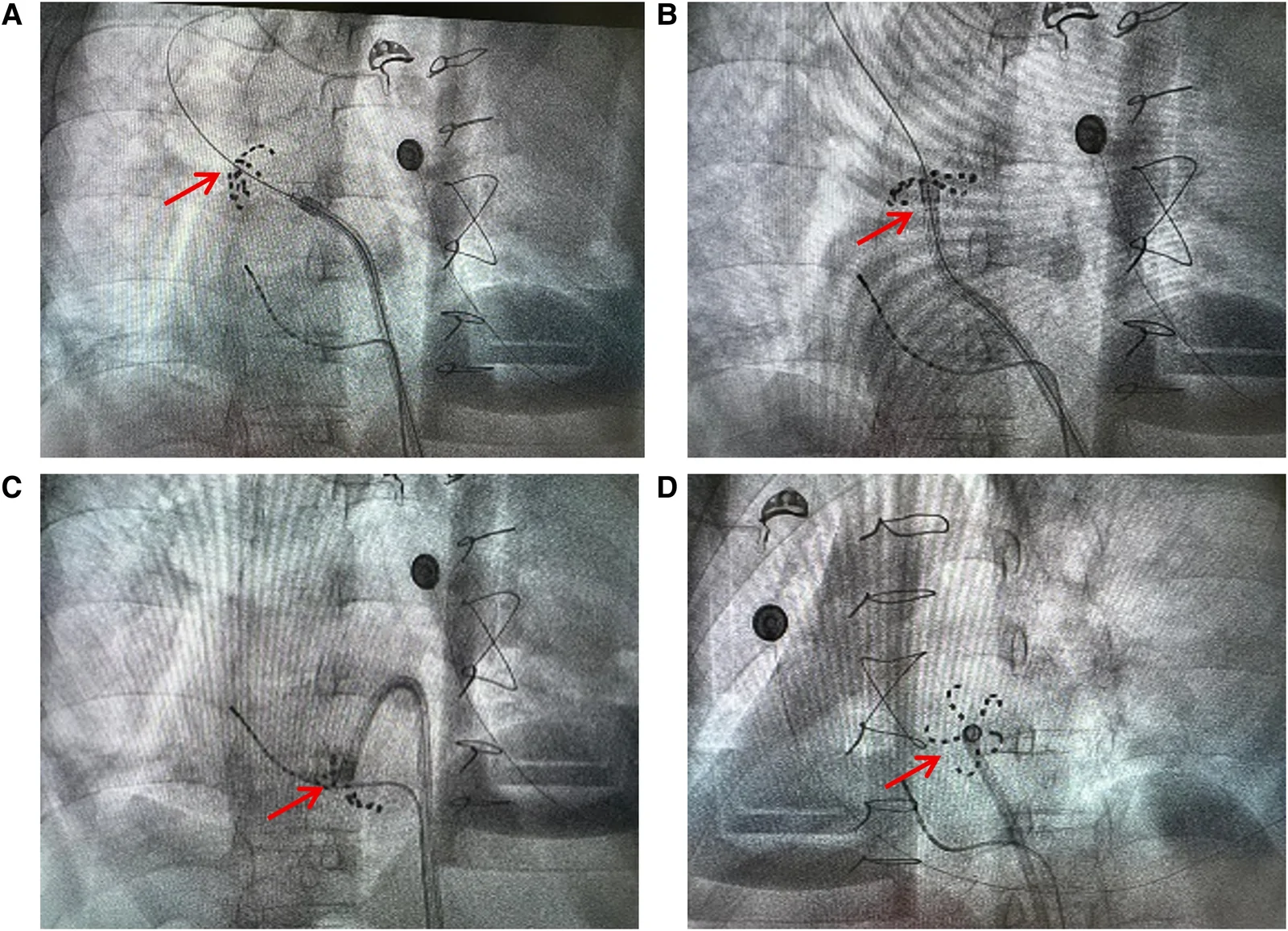
Fluoroscopic images demonstrating different configurations of the Farawave catheter during pulsed field ablation. (*A*) Right superior pulmonary vein isolation, right anterior oblique view, basket configuration. (*B*) Left atrial roof ablation, right anterior oblique view. (*C*) Right cavotricuspid isthmus ablation, right anterior oblique view. (*D*) Posterior wall ablation, left anterior oblique view, flower configuration. The arrows indicate different Farawave catheter positions.

Under fluoroscopic guidance and with adequate endocardial contact, PFA was performed. The catheter configuration was alternated between baskets (*Figure 4A*) and flower shapes until pulmonary vein (PV) potentials were eliminated on the electrogram.

In addition to pulmonary vein isolation (PVI), left atrial roof and posterior wall ablation were performed because of the patient’s prior atrial surgery and suspected atypical AFL substrate (*Figure 4B–D*). Cavotricuspid isthmus (CTI) ablation was added due to documented AFL on pre-procedural ECG recordings.

Pulsed field ablation was delivered in bipolar mode at 1.8 kV with a pulse duration of 2.5 s using basket and flower catheter configurations. Circumferential PVI was performed under fluoroscopic guidance with 12 applications in the right superior PV, 10 in the right inferior PV, 10 in the left inferior PV, and 12 in the left superior PV. Additional lesions were delivered to the left atrial roof, posterior wall, and CTI (two applications each). PVI was confirmed by elimination of PV potentials and demonstration of exit block during pacing manoeuvres. A bidirectional CTI block was achieved, with conduction time prolongation from 70 ms before ablation to 130 ms after ablation.

**Figure 5:**
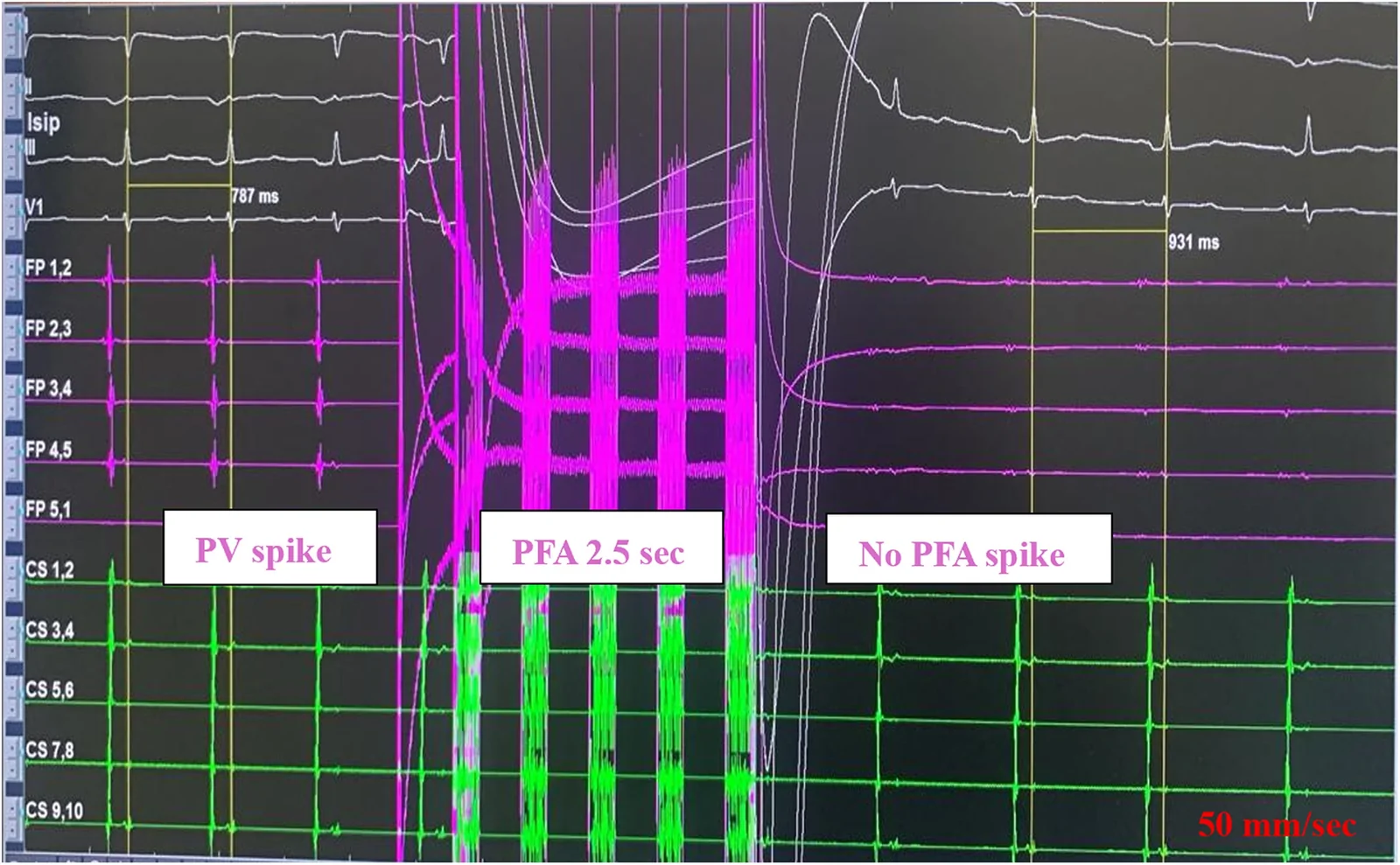
Electrocardiographic recordings obtained during single-shot pulmonary vein isolation using the Farapulse system. Electrograms demonstrate the elimination of pulmonary vein potentials after pulsed field ablation, confirming an entrance block consistent with successful pulmonary vein isolation. The coronary sinus (CS) catheter is positioned in the right ventricle for backup pacing and records ventricular electrograms.

All lesions were created using the Farapulse PFA system, aiming to achieve transmural lesions while maintaining safety in the mirror-image anatomy (*Figure 5*).

The skin-to-skin procedure time was 100 min. Total fluoroscopy time was 35,1 min. Post-procedure, transthoracic echocardiography was conducted, which excluded the presence of a significant pericardial effusion. The patient was monitored for 24 h post-procedure. The patient was discharged. The patient is now 6 months post-ablation without recurrence of AF and AFL.

## Discussion

Pulsed field ablation has demonstrated high efficacy and a favourable safety profile for AF ablation across multiple clinical studies, with consistently high rates of durable PVI and low incidence of procedure-related complications.^3–5^ More recent comparative studies have further shown that PFA is non-inferior to conventional thermal ablation techniques, including radiofrequency and cryoballoon ablation.^6,7^ These clinical results are largely due to the unique non-thermal mechanism of PFA, based on irreversible electroporation, which enables selective myocardial ablation with minimal damage to the surrounding heart tissue. This feature is particularly relevant in anatomically complex cases, where procedural safety and precision are critical.

In the present case, PFA was successfully used for the treatment of paroxysmal AF and AFL in a patient with dextrocardia and situs in vs. totalis. Despite the mirrored anatomy, PVI, left atrial roof, and posterior wall ablation, and CTI ablation were performed without complications, with no arrhythmia recurrence during 6 months of follow-up. This case highlights the feasibility of PFA not only for AF but also for AFL in complex congenital anatomy.

Data on PFA in dextrocardia remain limited. Scaglione *et al*. reported a technically challenging case requiring a transjugular approach because of an interrupted inferior vena cava, highlighting the importance of vascular anatomy in procedural planning.^8^ Chen *et al*.^9^ subsequently demonstrated the feasibility of a conventional transfemoral PFA approach in situs in vs. with dextrocardia, while Kikuchi *et al*.^10^ further confirmed the applicability of PFA for AF ablation in this rare anatomical setting. However, unlike the previously published reports focused primarily on PVI for AF, our case additionally demonstrates the feasibility of combined PFA treatment of both AF and typical AFL in the patient with prior surgical ASD patch repair. This is clinically relevant because previous atrial septal surgery may complicate transseptal access and catheter manipulation owing to altered septal anatomy and scarring. Therefore, our report expands the current experience not only by adding another anatomical variant but also by demonstrating the applicability of PFA in a more complex substrate requiring combined left- and right-sided atrial ablation.

The FARAPULSE PFA System (Boston Scientific) has demonstrated myocardial selectivity and consistent PVI.^11^ In our case, this system allowed efficient lesion delivery despite challenging spatial orientation.

Clinical studies using multi-electrode PFA catheters with flower- and basket-shaped configurations have demonstrated favourable safety and efficacy outcomes.^12^

Compared with cryoballoon ablation, PFA is associated with shorter procedural duration but may require longer fluoroscopy exposure, while maintaining similar periprocedural safety.^13,14^ This is consistent with our experience, where the total procedure time was 100 min.

Importantly, PFA was not limited to PVI in this case. Additional CTI ablation was performed for AFL, supporting previous observations that PFA can be effective in CTI-dependent circuits.^15^ Although data on PFA for AFL remain limited, our findings suggest that it may be a viable alternative to conventional thermal ablation for typical flutter.

A marked increase in troponin levels was observed after the procedure, with a peak value >15 ng/mL on the first post-procedural day, followed by a decline to 0.136 ng/mL within 72 h. Similar post-procedural biomarker elevations after PFA have been reported previously and are considered a procedural finding associated with myocardial electroporation.^4^ In our patient, no chest pain, electrocardiographic changes, haemodynamic instability, or echocardiographic evidence of pericardial complications were observed during follow-up. In addition, previous studies have demonstrated relative preservation of non-cardiac tissues, including nerves and smooth muscle, after PFA, which may contribute to its favourable safety profile.^5^

However, several limitations should be acknowledged. First, this is a single-case report, limiting the generalizability of the findings. Second, the follow-up duration was relatively short. Third, total fluoroscopy time was 35.1 min, relatively high, likely related to the complex mirror-image anatomy, previous congenital heart surgery, difficult CS cannulation, small body size requiring catheter exchange, and the absence of advanced imaging or mapping guidance. Increased fluoroscopy exposure remains an important consideration in anatomically complex procedures and may affect reproducibility across centres. Finally, the higher cost of PFA compared with conventional ablation techniques remains a limitation for broader clinical adoption.^14^

## Conclusion

This case demonstrates that combined PFA for AF and AFL is feasible in a patient with complex congenital anatomy, including dextrocardia after ASD repair, with pre-procedural cardiac CT playing a crucial role in anatomical orientation and procedural planning.

## Supplementary Material

ytag599_Supplementary_Data

## Data Availability

The data underlying this article will be shared on reasonable request to the corresponding author.

## Lead author biography

Professor Ayan Abdrakhmanov is an interventional arrhythmologist and Director of the Heart Rhythm Research Institute at Astana Medical University, as well as the Heart Rhythm Centre at the National Coordination Centre for Emergency Medicine in Astana, Kazakhstan. He specializes in the diagnosis and management of cardiac arrhythmias in both paediatric and adult populations. His clinical expertise encompasses advanced electrophysiological diagnostics, pharmacological therapy, catheter ablation, and implantation of cardiac devices, including implantable monitors, pacemakers (including leadless systems), subcutaneous cardioverter-defibrillators (S-ICD), cardiac resynchronization therapy (CRT) devices, and left atrial appendage occlusion (LAAO).

## Notes

**Consent:** The authors confirm that written consent to submit and publish this case report, including images and associated text, has been obtained from the patient in accordance with the COPE guidance.
