# Supplementary material for: Pulsed field ablation for paroxysmal atrial fibrillation and atrial flutter in a patient with dextrocardia and prior secundum atrial septal defect repair: a case report
Source: Eur Heart J Case Rep. 2026 Aug 11;10(8):ytag599. doi: 10.1093/ehjcr/ytag599 (PMC13492335; doi:10.1093/ehjcr/ytag599)
Supplement: Supplementary file 1 [file ytag599_supplementary_data.docx]

| **Date** | **Event** |
| --- | --- |
| 2014 | Surgical secundum ASD repair |
| 2025 | Progressive palpitations and symptomatic AF/AFL |
| Pre-procedure | CT imaging performed |
| Procedure day | Combined PFA for AF and AFL |
| 6 months | No arrhythmia recurrence on Holter monitoring |

**Table S1. The key clinical events.**
